# Never say “never the twain shall meet”: Combining antiretroviral therapy and RNA vaccine to obtain an adequate humoral immune response in people living with HIV

**DOI:** 10.1016/j.lanepe.2022.100310

**Published:** 2022-01-25

**Authors:** Jean-Pierre Routy, Stéphane Isnard

**Affiliations:** a Infectious Diseases and Immunity in Global Health Program, McGill University Health Centre, Research Institute, Montréal, QC, Canada; b Chronic Viral illness Services, McGill University Health Centre, Montréal, QC, Canada; c Department of hematology, McGill University Health Centre, Montréal, QC, Canada; d Canadian HIV trials network, Canadian Institutes for Health Research, Vancouver, BC, Canada

Development and worldwide access of safe and effective RNA-based SARS-CoV-2 vaccines against COVID-19 have garnered immense interest. Despite this scientific *tour de force*, the journey from vaccine discovery to global immunity against COVID-19 remains a significant challenge.

Host factors associated with reduced vaccine efficacy and durability are well established and include older age in adults, chronic inflammation, CMV latent infection and immunosuppression.^1^ These factors hamper vaccine response with limited elicitation of antibody titers and reduced T-cell response, in relation with low thymic output and a restricted naïve T-cell repertoire. In parallel, solid organ transplant recipients and patients receiving immuno-chemotherapies for cancer also presented a lower COVID-19 vaccine response.^2^^,^^3^

Information on COVID-19 vaccine immunogenicity and immune response durability in people living with HIV (PLWH) is not yet established, as these persons were mostly excluded from large prospective vaccine studies.

PLWH represent a distinctive group among immunosuppressed patients. HIV infection is characterized by a reduction of the number of CD4 T-cells, predominantly the mucosal protective CD4 T-helper 17 (Th17) cells that are rapidly infected and depleted from the digestive track and to a lessen extend from the respiratory track. Such immune damage alters epithelial barrier, resulting in microbial translocation and systemic inflammation that further induce T-cell activation. Although long-term antiretroviral therapy (ART) prompts CD4 T-cell recovery, some immune defects persist such as immune activation, low CD4/CD8 ratio and risk of developing inflammatory non-AIDS comorbidity. As cell-mediated immunity, including CD4 helper T-cells, has emerged as a critical contributor to the COVID-19 vaccine response, the influence of combined immunosuppression and immune activation in PLWH remains a poorly addressed question.

A handful number of studies have investigated responses to COVID-19 vaccines in PLWH. An observational study involving 14 PLWH on ART assessed the anti-Spike antibody titres following two doses of mRNA vaccines. Encouragingly, immunogenicity was not different from previous results in HIV-uninfected participants, suggesting that mRNA vaccination should succeed in PLWH with viral suppression, regardless of their CD4 T-cell count.^4^ However, a German study found lower anti-Spike IgG titres and neutralising activity 35 days after two doses of a mRNA vaccine in 55 ART-treated PLWH compared to controls.^5^

In this issue of *The Lancet Regional Health – Europe,* Lombardi et al. assessed in a single clinical site in Milan, Italy, the immunogenicity of the mRNA-1273 vaccine among PLWH.^6^ A total of 71 adult PLWH outpatients were prospectively enrolled in February 2021. Participants were on suppressive ART, with CD4 T-cell counts greater than 500 cells/μL. The two doses of mRNA-1273 vaccine were administrated 28 days apart. Anti-Spike antibody titres and neutralising antibody activity were used as a readout of immunogenicity, collected 28 days after the first and the second vaccine doses. Individuals without HIV, mostly health care workers, were recruited as controls. Early findings assessed a month after the second vaccine dose showed similar anti-Spike antibody titres and neutralising antibody activity in the two groups. Conversely to the line in the Rudyard Kipling poem: The Ballad of East and West “never the twain shall meet”, herein, the combination of the twain (two), ART and vaccine, led to an immunogenic response that was not different from HIV-uninfected control. This observation in PLWH on ART is very encouraging at least for the first two months following the first dose of vaccine.

However, the study did not address vaccine effectiveness and durability of humoral, nor cellular immune response, key factors for real life vaccine success. Recent data in French health care workers indicate that post-vaccination antibody titer decreases faster in vaccinated people than in COVID-19-infected individuals who then received vaccination.^7^ On the other hand, Alrubayyi et al. showed in London, UK, that antibody and specific T-cell responses to SARS-CoV-2 infection were comparable between PLWH and negative subjects, and persisted 5-7 months following mild COVID-19 disease. Importantly, the magnitude of specific T-cell responses was associated with the naïve CD4 T-cell pool size and the CD4/CD8 ratio in PLWH. Moreover, PLWH with CD4 T-cell count below 200 cells/μL, a threshold for increased risk of AIDS, may not respond well and durably to vaccine, as previously reported for other vaccines.^1^ These findings highlight the importance of CD4 T-cells in a successful vaccine response and that sub-optimal immune reconstitution, even on ART, could hinder vaccine responses to SARS-CoV-2.^5^^,^^8^

Vaccine response might, in any population, wanes due to developments of variants escaping T-cells and antibody selective immune pression (i.e.: antigenic drift)^9^ and to short-lived immune memory for SARS-CoV-2, which is not a virus with obligate viremic spread .

To address long-term benefit of COVID-19 vaccines in PLWH, we need to establish large prospective cohorts to assess level and durability of humoral and cellular immunogenicity to current and future variants, combined with their safety and tolerability.^10^

## Declaration of interests

The authors declare no conflict of interest.
